# The complete chloroplast genome of *Platycerium wallichii* (Polypodiaceae), an endangered and ornamental fern species 

**DOI:** 10.1080/23802359.2021.1950057

**Published:** 2021-07-09

**Authors:** Kaikai Wang, Jieqiu Duan, Yong Ding, Jianying Xiang, Hongmei Liu

**Affiliations:** a Xishuangbanna Tropical Botanical Garden, Chinese Academy of Sciences, Menglun, China; b University of Chinese Academy of Sciences, Beijing, China; c College of Life Science, Southwest Forestry University, Kunming, China; d Yunnan Academy of Biodiversity, Southwest Forestry University, Kunming, China

**Keywords:** Plastome, conservation, epiphytes, phylogenomics

## Abstract

The complete chloroplast genome of a staghorn fern species (*Platycerium wallichii*) was sequenced. The total genome was 158,286 bp in length, containing four regions: large single-copy (LSC) region (79,087 bp), small single-copy (SSC) region (21,397 bp), and two inverted repeat regions (IRs; 28,901 bp per each). In total 129 genes were annotated including 88 coding genes, 33 tRNAs, and 8 rRNAs. The overall GC content of the genome is 40.5%. Phylogenetic analysis supported the monophyly of both the subfamily Platycerioideae and the genus *Platycerium*. The genome data provides crucial information to support the future conservation and horticulture research.

*Platycerium wallichii* Hook. is an epiphytic fern belonging to the subfamily Platycerioideae of Polypodiaceae (PPGI 2016), it is distributed in tropical lowland rainforest, occurring in China, India, Malaysia, Myanmar, and Thailand (Zhang and Gilbert 2013). This species as well as its congeneric species is usually found on tree trunks forming a very impressively splendid view in the field, therefore *Platycerium* is popular ornamental fern in flower markets, which has resulted in an increasing loss of wild populations. *Platycerium wallichii* is considered to be endangered in China by deforestation and loss of habitat, it was classified as a national second-class protected wild plant in the Information System of Chinese Rare and Endangered Plants (ISCRPF) (http://www.iplant.cn/rep) and Critically Endangered by IUCN (https://www.iucn.org/resources/conservation-tools/iucn-red-list-threatened-species).

In this study, we report the complete chloroplast genome of *P. wallichii* for the first time. Fresh leaf material was collected from Yingjiang, Yunnan, China (24°45′01″N, 97°60′17″E). The specimen was deposited at the Herbarium of Xishuangbanna Tropical Botanical Garden, CAS (HITBC, http://www.xtbg.ac.cn/jgsz/zcxt/rdzwzzzyk/; Hongmei Liu, liuhongmei@xtbg.ac.cn) under the voucher number L2691/CP06). Genomic DNA was extracted from 2 g leaves using the CTAB method (Doyle and Doyle 1987), 0.5 ug DNA was fragmented to reconstruct short-insert (150 bp) libraries following the manufacturer’s manual (Illumina) and then used for sequencing. The DNA sample was indexed by tags and pooled together in one lane of a Genome Analyzer (Illumina HiSeq 2000) for sequencing at BGI-Shenzhen, and >4.0 Gb of reads were obtained. GetOrganelle toolkit (Jin et al. 2020) and Geneious (https://www.geneious.com) were employed to assemble and annotate the genome. The reference genomes identified as *P. bifurcatum* for annotation were MN623367 and MW042261. The newly sequenced and annotated plastid genome was submitted to the GenBank (accession number MW467509) and open to the public.

The chloroplast genome of *P. wallichii* has the quadripartite structure, with the total length is 158,286 bp including a large single-copy (LSC) region of 79,087 bp, a small single-copy (SSC) region of 21,397 bp, and a pair of inverted repeats regions (IR) of 28,901bp. GC content of the genome is 40.5%. The chloroplast genome contains 129 genes including 88 protein-coding genes, 33 tRNAs, and eight rRNA genes. Comparing the plastomes among the congeneric species, there is a sequence length variation within the genus with *P. bifurcatum* having both 160,442 bp (MW042261) and 156,985 bp (MN623367) in length with *P. wallichii* (158,286 bp) sitting in the middle.

The genome sequence of *P*. *wallichii* was incorporated into a matrix including 26 taxa covering five subfamilies of Polypodiaceae (PPGI 2016) with the aim to reconstruct the infra-familial phylogenetic relationship. 80 coding genes were selected, aligned, and concatenated into a single matrix using MAFFT (Katoh and Standley 2013). The phylogenetic analyses were constructed using RAxML version 8.2.12 (Stamatakis 2014) with Maximum Likelihood method, nucleotide substitution model of GTR + Gamma was used with 1000 bootstrap replicates. *Loxogramme lankokiensis* (Loxogrammoideae) was selected as outgroup according to Schneider et al. (2004). Monophyly of the subfamily Platycerioideae as well as the two genera *Platycerium* and* Pyrrosia* (Figure 1) was strongly supported. The genome of *P. wallichii* was nested between the two samples submitted as *P. bifurcatum* therefore we suspect one of the *P. bifurcatum* was misidentified.

**Figure 1.**
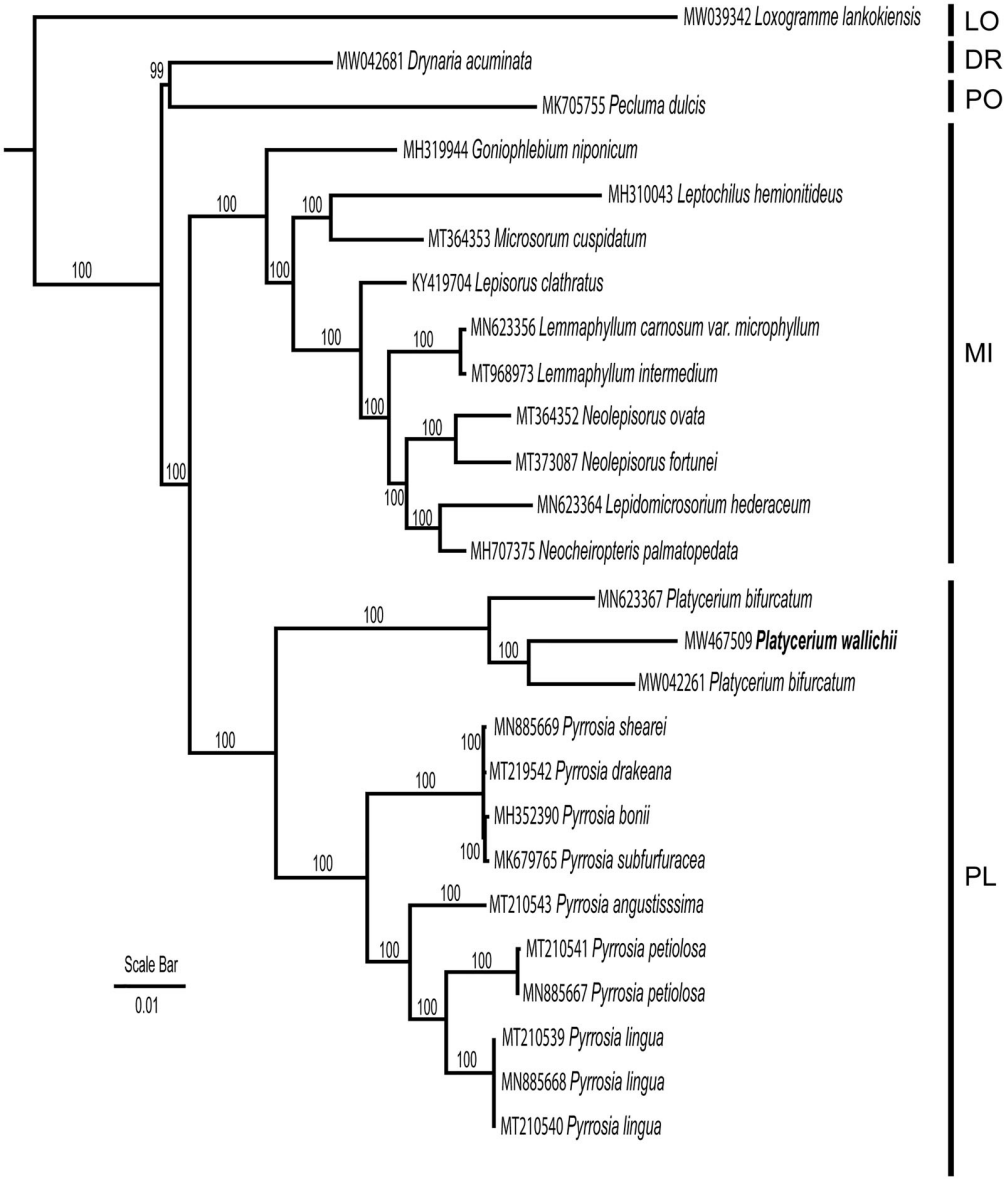
Maximum likelihood phylogeny reconstructeded from 26 chloroplast genomes by RAxML. The sampling covered representatives of five subfamilies of Polypodiaceae. *Loxogramme lankokiensis* was selected as outgroup. LO: Loxogrammoideae; DR: Drynarioideae; PO: Polypodioideae; MI: Microsoroideae; PL: Platycerioideae.

The newly generated plastome of *P. wallichii* provides useful information for future phylogeny, conservation, and horticulture research, and will allow us to have a better understanding of the phylogenetic relationships for the derived fern family Polypodiaceae.

## Data Availability

The genome sequence data of *Platycerium wallichii* that support the findings of this study are openly available in GenBank of NCBI at (https://www.ncbi.nlm.nih.gov/nuccore/MW467509.1) under the accession no. MW467509.. The associated Bioproject, Sequence Read Archive (SRA), and Biosample numbers are PRJNA737298, SRR14812826, and SAMN19689021, respectively.
